# Editorial: Let There Be Light: Biological Impact of Light Exposure in the Laboratory and the Clinic

**DOI:** 10.3389/fneur.2018.00851

**Published:** 2018-10-09

**Authors:** Monica M. C. Gonzalez, Diego A. Golombek

**Affiliations:** ^1^Division of Sleep and Chronobiology, Instituto Ferrero de Neurología y Sueño, Buenos Aires, Argentina; ^2^Department of Science and Technology, Universidad Nacional de Quilmes, Bernal, Argentina

**Keywords:** circadian system, desynchronization, retina, sleep, constant light/dark, metabolism

Light is a relevant environmental factor due to its role in the synchronization of mammalian biological rhythms driven by the master circadian clock, the suprachiasmatic nucleus (SCN) (1–4). Although light exposure has obvious beneficial properties on the quality of life, different articles have highlighted the deleterious effects of certain artificial lighting conditions that might affect the health and wellbeing of human beings and laboratory animals. Indeed, we are exposed to artificial light during the night and, in addition, constant lighting conditions or altered photoperiods are tools frequently used in experimental animals, neglecting the fact that these environments might influence the integrity of the nervous system and endocrine, physiological and behavioral-dependent processes that rely on the activity of the biological clock.

The articles in this research topic aim at providing the reader with an updated overview assessing the impact of altered light exposure in retinal integrity and the adverse outcomes on health attributed to commonly-used lighting conditions in modern society and experimental settings.

In the open article, “*Non-Visual Photopigments Effects of Constant Light-Emitting Diode Light Exposure on the Inner Retina of Wistar Rats*” Benedetto et al. provide new data on the effect of light pollution on retinal degeneration, evidencing a differential photoresistent mechanism among different retinal cell populations when exposed to constant light. Rats without pupil dilatation and under constant exposure to 200 lux of diffuse LED light for several days exhibit induced photoreceptor cell death only in the outer nuclear layer. Moreover, the localization and protein expression of non-visual photopigments melanopsin (OPN4) and neuropsin (OPN5) responsible of sensing light in the inner retina are modified, although the inner structure and retinal ganglion cells (RGCs) survival stay unaffected.

The paper “*In a Heartbeat: Light and Cardiovascular Physiology*” by Chellappa et al. describes the network involved in the putative role of the SCN as mediator of cardiovascular physiology, and review the role of light control on the cardiovascular system, besides emphasizing the contribution of certain light modalities to adverse cardiovascular events. The participation of different networks of peripheral oscillators driven by SCN outputs in metabolism and energy expenditure are reviewed in “*Circadian and Metabolic Effects of Light: Implications in Weight Homeostasis and Health*” by Plano et al.. The authors discuss the way in which abnormal LD cycles frequently used in experimental protocols or urban environments may lead to cardiometabolic diseases, obesity, and diabetes by altering these circadian metabolic feedback loops. The article by Fisk et al.. “*Light and Cognition: Roles for Circadian Rhythms, Sleep, and Arousal*,” expands these findings to the cognitive sphere, and describes the mechanisms mediating photoentrainment and the way in which light regulates sleep, arousal, and different cognitive process in humans and laboratory species. Indeed, as the authors demonstrate, abnormal LD cycles induce changes in sleep and wake, which in turn have a profound effect on cognition. The common theme here is that the use of unified LD protocols is fundamental to compare physiological and behavioral mechanisms in any kind of experimental setting.

Finally, in “*Dim Light at Night and Constant Darkness: Two Frequently used Lighting Conditions that Jeopardize the Health and Wellbeing of Laboratory Rodents”*, González reviews the adverse outcomes attributed to commonly used lighting conditions in animal facilities. Light exposure at night and constant dim light or darkness have detrimental short- and long-term effect in rodents that are reflected in disrupted circadian rhythms, neural death, depressive-behavioral phenotype, and metabolic, physiological, and synaptic plasticity deregulation. Tips to measure light intensity and recommendations are also provided to ensure the welfare of laboratory animals and increase the likelihood of producing reliable and reproducible results.

In summary, light is probably one of the main factors that has sculpted the evolution of life on Earth. While some disciplines do take adequate care of its use and potential harm, the effects of artificial lighting and photoperiodic schedules (including extreme protocols such as constant light or darkness) needs to be acknowledged by all researchers using humans or animal models, since the outcome of an experiment—as well as the health and quality of life of such models—might be severely affected or compromised by such environmental conditions. This special topic represents a series of observations and recommendations in this respect. In animal science, light can be friend or foe, depending on its use and control.

## Author contributions

MG conceived the project. MG and DG commissioned, selected, and edited the proposals and wrote the editorial.

### Conflict of interest statement

The authors declare that the research was conducted in the absence of any commercial or financial relationships that could be construed as a potential conflict of interest.
